# Worldwide burden of liver cancer due to metabolic dysfunction-associated steatohepatitis from 1990 to 2019: insights from the Global Burden of Disease study

**DOI:** 10.3389/fonc.2024.1424155

**Published:** 2024-08-29

**Authors:** Minshan Huang, Hang Chen, Hui Wang, Xianmei Wang, Da Wang, Yu Li, Qingqing Zhou, Dan Zhang, Mengwei Li, Lanqing Ma

**Affiliations:** The First Affiliated Hospital, Yunnan Institute of Digestive Disease, Kunming Medical University, Kunming, China

**Keywords:** global burden of disease, hepatocellular carcinoma, metabolic dysfunction-associated steatohepatitis, observational study, population study

## Abstract

**Introduction:**

Metabolic dysfunction-associated steatohepatitis (MASH) is increasingly becoming a prevalent cause of hepatocellular carcinoma (HCC). Our study examines the burden of MASH-related HCC globally, regionally, and nationally, along with associated risk factors from 1990 to 2019, considering variables such as age, sex, and socioeconomic status.

**Objective:**

We aimed to report the global, regional, and national burden of liver cancer due to MASH and its attributable risk factors between 1990 and 2019, by age, sex, and sociodemographic index (SDI).

**Methods:**

Utilizing the Global Burden of Disease 2019 project, we analyzed data on prevalence, mortality, and disability-adjusted life years (DALYs) for liver cancer attributable to MASH across 204 countries. We provided counts and rates per 100,000 population, including 95% uncertainty intervals.

**Results:**

In 2019, there were 46.8 thousand cases of MASH-related HCC, leading to 34.7 thousand deaths, and 795.8 thousand DALYs globally. While the prevalence increased by 19.8% since 1990, the death and DALY rates decreased by 5.3% and 15.1%, respectively. The highest prevalence was in High-income Asia Pacific, with the greatest increases observed in Australasia, Central Asia, and High-income North America. Southern Sub-Saharan Africa reported the highest death rate, while the lowest rates were in parts of Latin America, Central Sub-Saharan Africa, and Eastern Europe. DALY rates were the highest in Southern Sub-Saharan Africa and the lowest in Tropical Latin America.

**Discussion:**

The burden of MASH-related HCC is expected to rise slightly over the next decade. This disease, which is not associated with the SDI, remains a major public health problem. In addition, the escalating rates of obesity, demographic shifts, and an aging population could position MASH as a leading factor in liver cancer cases, surpassing viral hepatitis. It is imperative, therefore, that the forthcoming years see the implementation of strategic interventions aimed at the early detection and prevention of liver cancer associated with MASH.

## 1 Introduction

Nonalcoholic steatohepatitis (NASH) is characterized by hepatic steatosis of over 5%, accompanied by inflammation, and may or may not involve fibrosis. It exhibits a close, bidirectional relationship with the components of metabolic syndrome (1, 2). Experts recommend using metabolic dysfunction-associated steatohepatitis (MASH) instead of NASH (3, 4). As a more severe variant of metabolic-associated fatty liver disease (MAFLD), MASH presents with lipid accumulation within the liver, coupled with inflammation and hepatic damage.

Approximately 60% of the patients with MAFLD progress to MASH. In the studies that were analyzed, it was found that approximately 40% of individuals with MASH experienced progression to fibrosis. Additionally, it was estimated that approximately 25% of patients with MASH worldwide advanced to more severe stages of liver disease, including cirrhosis and hepatocellular carcinoma (HCC) (5).

HCC has escalated to a major public health concern globally, positioned as the sixth most widespread cancer with annual case counts exceeding 900,000. It remains the third most common cause of cancer-induced fatalities, accounting for over 800,000 deaths each year (6). Previously, hepatitis C virus (HCV) was thought to be the leading cause of HCC (7), but recent reports showed that up to 50% of newly diagnosed patients with HCC have non-viral HCC (8, 9). Over the next decade, the proportion of liver cancer cases due to MAFLD is expected to increase substantially. This increase is mainly due to the global epidemic trends of obesity and type 2 diabetes, both of which are major risk factors for MAFLD. The development of HCC from MASH is linked to a variety of complex factors, including cellular adaptability, ongoing inflammation, apoptosis, disruptions in the normal cell cycle, and various forms of cell death (10).

There is mounting evidence indicating that MASH plays a significant role in the development of HCC, making it an increasingly common cause of HCC worldwide. It is estimated that approximately 10%–30% of individuals with MAFLD progress to cirrhosis, and in the United States alone, approximately 6 million people are affected by MASH. Several cohort studies have demonstrated that over one-fourth of MASH-related HCC cases can occur without cirrhosis, a proportion significantly higher than what is observed in other liver diseases (11).

From 1990 to 2019, the Global Burden of Disease (GBD) study reported the burden of MASH-related HCC in 204 nations and territories, stratified by sex, region, and sociodemographic index (SDI). By analyzing the latest GBD data, researchers can establish more effective prevention strategies. The GBD repository has been instrumental for a myriad of studies in evaluating liver cancer’s incidence, prevalence, and mortality rates. Yet, there remains a notable gap in comprehensive global analyses focused on HCC trends specifically attributable to MASH, underscoring a pivotal area for future research endeavors. In this study, we report the prevalence, deaths, and DALYs associated with MASH-related HCC, and disease trend by age and sex in 204 countries and territories between 1990 and 2019, using data derived from the GBD 2019.

## 2 Methods

### 2.1 Data sources

Recognized as one of the most expansive and methodical epidemiological analyses globally, the GBD project offers an in-depth evaluation of a wide array of diseases and injuries, alongside numerous risk factors. The GBD 2019 study, published in 2020, provided a thorough assessment of 369 diseases and injuries, along with 87 risk factors across the globe (12, 13). From the GBD 2019 repository, we extracted global mortality and population data specific to HCC linked to MASH, further delineating these data by variables such as age, sex, year, nation, and region. The GBD 2019 data can be accessed through web-based tools (https://vizhub.healthdata.org/gbd-results/). This study complies with the Guidelines for Accurate and Transparent Health Estimates Reporting.

### 2.2 Disease definition

Experts proposed renaming nonalcoholic fatty liver disease (NAFLD) and nonalcoholic steatohepatitis (NASH) to metabolic-associated fatty liver disease (MAFLD) and metabolic dysfunction-associated steatohepatitis (MASH) (4). However, MASH outcomes were not yet included in the 2019 GBD database; therefore, we used the NASH data from the 2019 GBD database.

### 2.3 Sociodemographic index

The SDI extracted from the GBD 2019 data resources serves as a multidimensional gauge of a region’s welfare, incorporating elements like fertility rates in individuals under 25, per-capita income, and the educational level for those over 15. This index scales from 0 to 1, where higher figures denote a superior socioeconomic status (12).

### 2.4 Risk factors

Strong evidence indicates that the risk factors that cause liver cancer due to MASH are smoking status and high fasting plasma glucose. The proportion of DALYs that were attributable to each MASH-related HCC factor was also reported.

### 2.5 Statistical analysis

In our analysis, we illustrate the age-standardized rates, including the prevalence of MASH-related HCC and the associated DALYs, across 21 geographical regions and 204 countries. This presentation also extends to the examination of prevalence rates and counts categorized by age and sex. To explore the relationship between the SDI and the age-standardized prevalence/DALYs related to MASH-related HCC from 2005 to 2019, we employed a Gaussian process regression model. This advanced statistical technique allowed us to analyze how SDI correlates with age-adjusted prevalence and DALYs across various locales.

To enhance the prediction of ASMR and the anticipated number of deaths in the upcoming decade, our analysis employed the BAPC model. This approach proved more efficacious in forecasting outcomes compared to other models, such as Joinpoint regression and Poisson regression (14). The BAPC model, utilizing the Integrated Nested Laplace Approximation for analysis, is designed to ensure the smoothness of trend data across age, period, and cohort groups. It achieves this by assuming that the averages of the second-order differences across all effects follow an independent normal distribution. This assumption helps in moderating the fluctuation in parameters across adjacent time periods, thus ensuring a more stable and reliable prediction (14, 15). All data processing, analysis, and visualization in this study were accomplished by the R program (version 4.3.1).

## 3 Results

### 3.1 The overall burden of MASH-related HCC

#### 3.1.1 Global level

**Figure 1:**
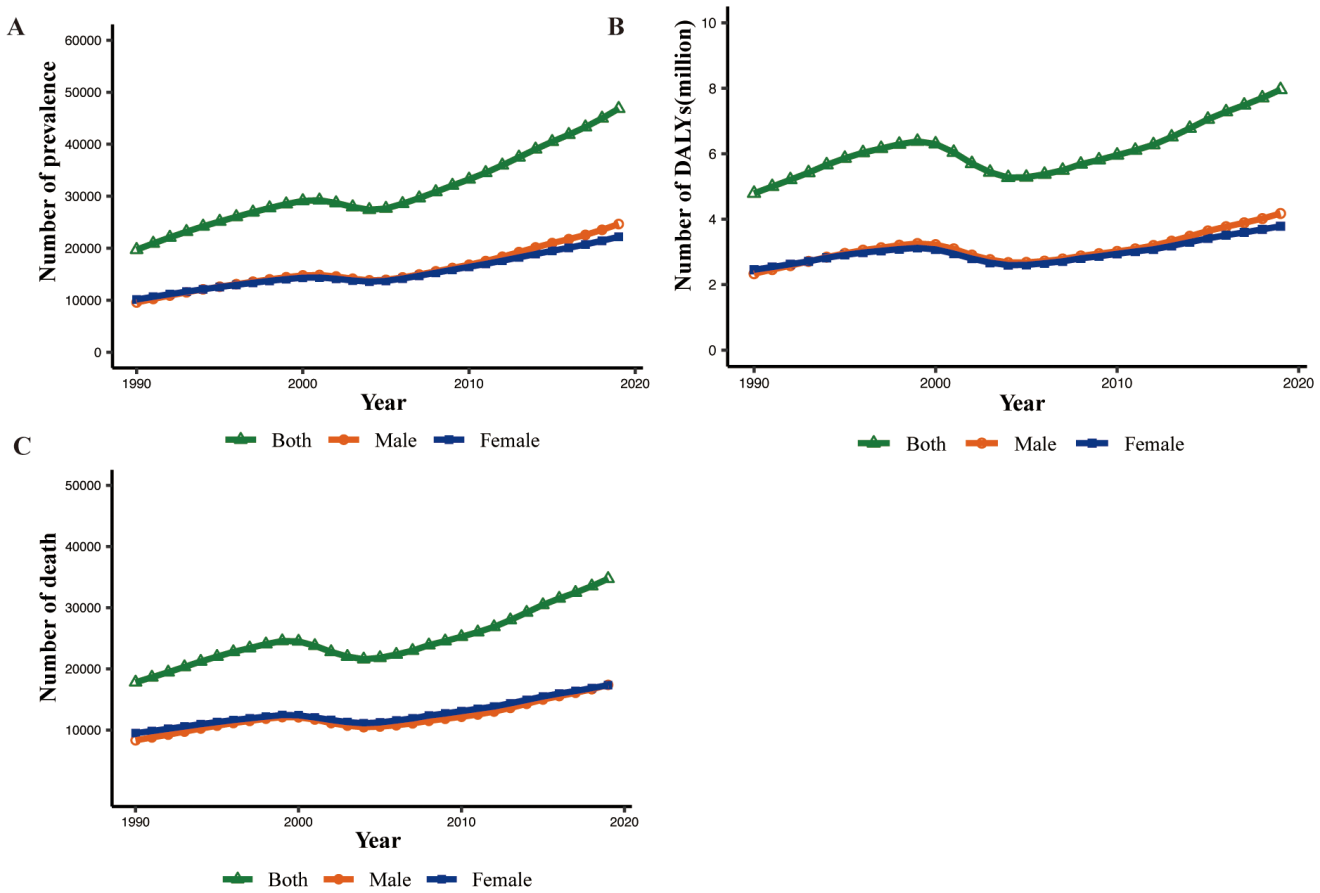
Global prevalence, death, and DALY numbers of MASH-related HCC for male, female, and both sexes from 1990 to 2019. **(A)** Prevalence. **(B)** DALYs. DALY, disability-adjusted life year. **(C)** Death.

In 2019, there were 46.8 thousand prevalent cases of MASH-related HCC globally, with an age-standardized point prevalence of 0.6 per 100,000, an increase of 19.8% since 1990. MASH-related HCC accounted for 34.7 thousand deaths in 2019, with an age-standardized rate of 0.4, a decrease of 5.3% since 1990. In 2019, the number of DALYs for MASH-related HCC globally was 795.8 thousand, with an age-standardized rate of 9.6 DALYs per 100,000, a 15.1% decrease since 1990 (**Table 1**). Although the changes in standardized rates had increase or decrease values, the absolute values of the three indicators generally increased from 1990 to 2019 (**Figures 1A–C**).

**Table 1:** Global prevalence, deaths, and DALYs attributable to MASH for both sexes combined in 2019 and percentage change from 1990 to 2019.

	Prevalence (95% UI)			Deaths (95% UI)			DALYs (95% UI)		
	No., in thousands (95% UI)	ASRs per 100,000 (95% UI)	Percentage change in ASRs from 1990 to 2019	No., in thousands (95% UI)	ASRs per 100,000 (95% UI)	Percentage change in ASRs from 1990 to 2019	No., in thousands (95% UI)	ASRs per 100,000 (95% UI)	Percentage change in ASRs from 1990 to 2019
Global	46.8 (38.2,57.6)	0.6 (0.5,0.7)	19.8 (4.8,36.3)	34.7 (28.4,43.2)	0.4 (0.4,0.5)	−5.3 (−16.7,7.2)	795.8 (657.3,975.8)	9.6 (8,11.8)	−15.1 (−26.1,−2.6)
High-income Asia Pacific	6.7 (5.1,8.6)	1.5 (1.2,1.9)	80.3 (51.4,114)	2.5 (1.9,3.2)	0.5 (0.4,0.6)	6.4 (−7.1,19.6)	42.3 (32.8,53.8)	10 (7.9,12.7)	−9.4 (−20.3,2.9)
High-income North America	4.5 (3.5,5.7)	0.8 (0.6,1)	207 (156.9,263)	2.9 (2.4,3.5)	0.5 (0.4,0.5)	127.6 (103.7,146.8)	58.6 (47.5,70.8)	9.8 (8,11.8)	128.5 (103.7,148.8)
Western Europe	4.3 (3.2,5.9)	0.5 (0.4,0.7)	137.2 (104.3,175.7)	2.8 (2,3.7)	0.3 (0.2,0.4)	53.1 (41.7,65.4)	48.9 (36.6,65.8)	5.8 (4.4,7.8)	47.4 (36.1,59.2)
Australasia	0.3 (0.2,0.4)	0.6 (0.5,0.9)	265.1 (189.8,365.1)	0.2 (0.2,0.3)	0.4 (0.3,0.6)	190.8 (150.3,231.1)	4.4 (3.2,5.9)	9.6 (7.1,12.6)	175.7 (135.8,216.3)
Andean Latin America	0.2 (0.1,0.2)	0.3 (0.2,0.4)	−16.8 (−35.8,6.4)	0.2 (0.1,0.3)	0.4 (0.2,0.5)	−16.2 (−34.3,6.5)	3.9 (2.7,5.5)	7 (4.8,9.9)	−23 (−40.9,−1.2)
Tropical Latin America	0.4 (0.3,0.4)	0.2 (0.1,0.2)	39.5 (31.7,48.2)	0.4 (0.3,0.4)	0.2 (0.1,0.2)	34.7 (26.6,43.2)	8.7 (7.4,10.2)	3.6 (3.1,4.2)	29.4 (21.7,37.3)
Central Latin America	0.8 (0.6,1)	0.3 (0.3,0.4)	29 (11.7,48.9)	0.8 (0.6,1)	0.3 (0.3,0.4)	24.8 (8,44.1)	17.4 (13.6,22.5)	7.3 (5.7,9.4)	19.7 (2.9,38.7)
Southern Latin America	0.2 (0.1,0.3)	0.2 (0.2,0.3)	100.7 (56.2,160.2)	0.2 (0.1,0.3)	0.2 (0.2,0.3)	83.8 (61.5,111.9)	3.9 (2.7,5.5)	4.7 (3.3,6.6)	76.7 (54.2,103.2)
Caribbean	0.2 (0.1,0.2)	0.3 (0.2,0.4)	−37.2 (−47.8,−24.4)	0.2 (0.1,0.2)	0.3 (0.2,0.5)	−40.7 (−50.4,−29.4)	3.5 (2.4,5)	6.8 (4.7,9.7)	−40.5 (−50.7,−28.3)
Central Europe	0.6 (0.4,0.8)	0.3 (0.2,0.4)	−26.6 (−37.2,−14)	0.6 (0.4,0.8)	0.3 (0.2,0.4)	−35.5 (−45,−25.1)	11.6 (8.4,16.1)	5.7 (4.1,7.8)	−34.4 (−44.3,−22.6)
Eastern Europe	0.8 (0.6,0.9)	0.2 (0.2,0.3)	124.2 (99.9,151.1)	0.8 (0.7,1)	0.2 (0.2,0.3)	114.3 (92.2,139.3)	17.5 (14.5,21.5)	5.4 (4.5,6.5)	110.4 (86.4,136.6)
Central Asia	0.4 (0.3,0.6)	0.6 (0.4,0.8)	256.8 (200.1,318.3)	0.4 (0.3,0.6)	0.7 (0.5,0.9)	260.6 (204.8,325.1)	11.2 (7.8,15.8)	14.6 (10.4,20.5)	243.8 (188.1,304.5)
North Africa and Middle East	3.6 (2.6,5.1)	0.8 (0.6,1.2)	69.2 (29.6,127)	2.7 (1.9,3.9)	0.7 (0.5,0.9)	28.4 (−2,75.8)	70.4 (47.5,104)	15.2 (10.5,22.2)	27.9 (−2.9,76.4)
South Asia	4.1 (3.4,5.1)	0.3 (0.2,0.4)	16.1 (−4.9,38.7)	4.2 (3.4,5.1)	0.3 (0.3,0.4)	15 (−7.3,37.4)	104.7 (84.5,127.9)	7.1 (5.7,8.6)	12 (−8.3,33)
Southeast Asia	4.4 (3.1,6.1)	0.7 (0.5,1)	51.5 (19.6,92.3)	4.1 (2.9,5.6)	0.7 (0.5,1)	42.1 (12.6,80.4)	96.2 (68.8,131.5)	15.6 (11.2,21.4)	28.8 (2.1,62.9)
East Asia	13.4 (10.4,16.7)	0.6 (0.5,0.8)	−37.9 (−50.7,−22.5)	9.8 (7.9,12.1)	0.5 (0.4,0.6)	−53.5 (−62.4,−42.3)	238.3 (190.1,295.7)	11.4 (9.2,14)	−57.7 (−66.1,−47)
Oceania	0 (0,0)	0.3 (0.2,0.4)	7.1 (−11.9,31.5)	0 (0,0)	0.3 (0.2,0.5)	6.8 (−11.9,31.4)	0.5 (0.4,0.8)	7.1 (4.9,10.2)	3 (−15.5,27.2)
Western Sub-Saharan Africa	0.8 (0.6,1.1)	0.4 (0.3,0.6)	8.6 (−12.2,33.7)	0.8 (0.6,1.1)	0.5 (0.4,0.7)	9.8 (−10.6,35.3)	21.9 (16.2,29.2)	10.7 (8,14.4)	6.1 (−14,30.4)
Eastern Sub-Saharan Africa	0.6 (0.5,0.9)	0.4 (0.3,0.5)	21.1 (−1.4,49.7)	0.6 (0.5,0.8)	0.4 (0.3,0.6)	22.1 (2.7,48.4)	17.3 (12.4,23.3)	9.4 (6.7,12.6)	17.6 (−5.9,46.9)
Central Sub-Saharan Africa	0.1 (0.1,0.2)	0.2 (0.1,0.2)	3.1 (−22.7,37.4)	0.1 (0.1,0.1)	0.2 (0.1,0.3)	1.8 (−20.2,31.9)	2.8 (1.9,4.2)	4.2 (2.8,6.2)	0.6 (−25,35.5)
Southern Sub-Saharan Africa	0.4 (0.4,0.5)	0.8 (0.6,0.9)	22.1 (−27.4,81.7)	0.4 (0.4,0.5)	0.8 (0.6,1)	22 (−26.6,83.2)	11.9 (9.6,14.5)	19.1 (15.5,23.2)	20.6 (−27.6,79.7)

#### 3.1.2 Regional level

In 2019, High-income Asia Pacific (1.5) had the highest age-standardized point prevalences for MASH-related HCC (per 100,000), whereas Tropical Latin America (0.2), Central Sub-Saharan Africa (0.2), Southern Latin America (0.2), and Eastern Europe (0.2) had the lowest. Southern Sub-Saharan Africa (0.8), Southeast Asia (0.7), North Africa and Middle East (0.7), and Central Asia (0.7) had the highest age-standardized death rates from MASH-related HCC in 2019, with the lowest rates in Tropical Latin America (0.2), Central Sub-Saharan Africa (0.2), Eastern Europe (0.2), and Southern Latin America (0.2). In 2019, Southern Sub-Saharan Africa (19.1), Southeast Asia (15.6), and North Africa and Middle East (15.2) had the highest age-standardized DALY rates (per 100,000), whereas Tropical Latin America (3.6), Central Sub-Saharan Africa (4.2), and Southern Latin America (4.7) had the lowest (**Table 1**).

The largest increases in the age-standardized point prevalence of MASH-related HCC, from 1990 to 2019, were found in Australasia (265.1%), Central Asia (256.8%), and High-income North America (207%), with the greatest decreases in East Asia (−37.9%), the Caribbean (−37.2%), and Central Europe (−26.6%). In the same period, the largest increases in the age-standardized death rates of MASH-related HCC were found in Central Asia (260.6%), Australasia (190.8%), and High-income North America (127.6%), with the largest decreases in East Asia (−53.5%), the Caribbean (−40.7%), and Central Europe (−35.5%). The largest increases in the age-standardized DALY rates of MASH-related HCC were found in Central Asia (243.8%), Australasia (175.7%), and High-income North America (128.5%), with the largest decreases in East Asia (−57.7%), the Caribbean (−40.5%), and Central Europe (−34.4%) (**Table 1**).

#### 3.1.3 National level

**Figure 2:**
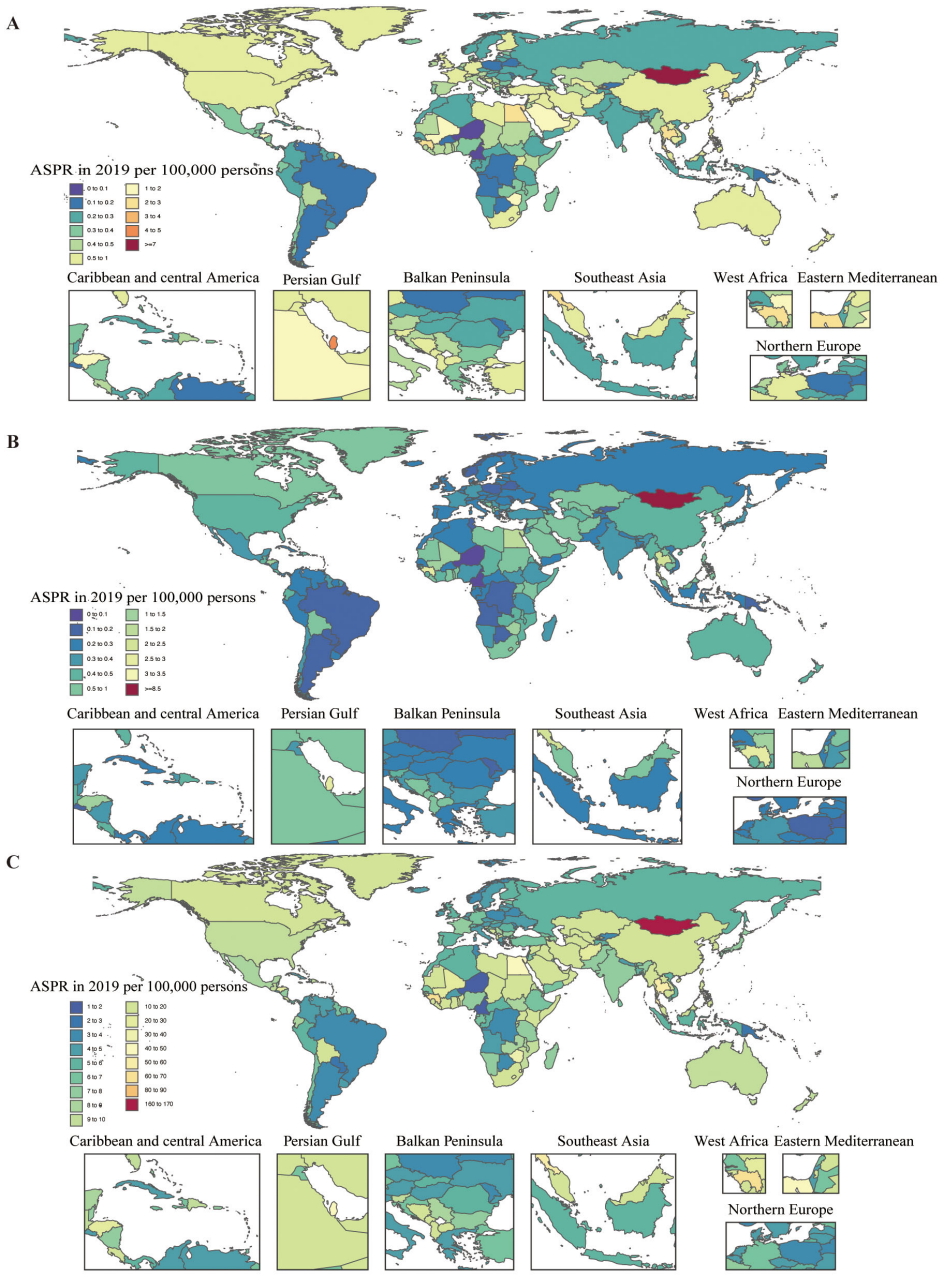
Age-standardized prevalence, death, and DALY rates attributable to MASH for both sexes combined in 2019. **(A)** Prevalence. **(B)** Deaths. **(C)** DALYs. DALY, disability-adjusted life year.

In 2019, the national age-standardized point prevalence of MASH-related HCC ranged from 0.05 to 7.01 cases per 100,000. Mongolia (7.01), Qatar (4.27), and Gambia (3.20) had the highest age-standardized point prevalences of MASH-related HCC, with Niger (0.05), Cameroon (0.06), and Papua New Guinea (0.10) having the lowest estimates (**Figure 2**). The national age-standardized death rates for MASH-related HCC in 2019 varied from 0.05 to 8.72 deaths per 100,000. The highest rates were seen in Mongolia (8.72), Gambia (3.43), and Guinea (2.75), whereas the lowest rates were found in Niger (0.05), Cameroon (0.07), and Paraguay (0.12) (**Figure 2**). In 2019, the national age-standardized DALY rate of MASH-related HCC ranged from 1.15 to 167.20 per 100,000. The highest rates were seen in Mongolia (167.20), Gambia (82.59), and Guinea (62.30), whereas the lowest rates were in Niger (1.15), Cameroon (1.60), and Papua New Guinea (2.49) (**Figure 2**).

### 3.2 Age and sex patterns

**Figure 3:**
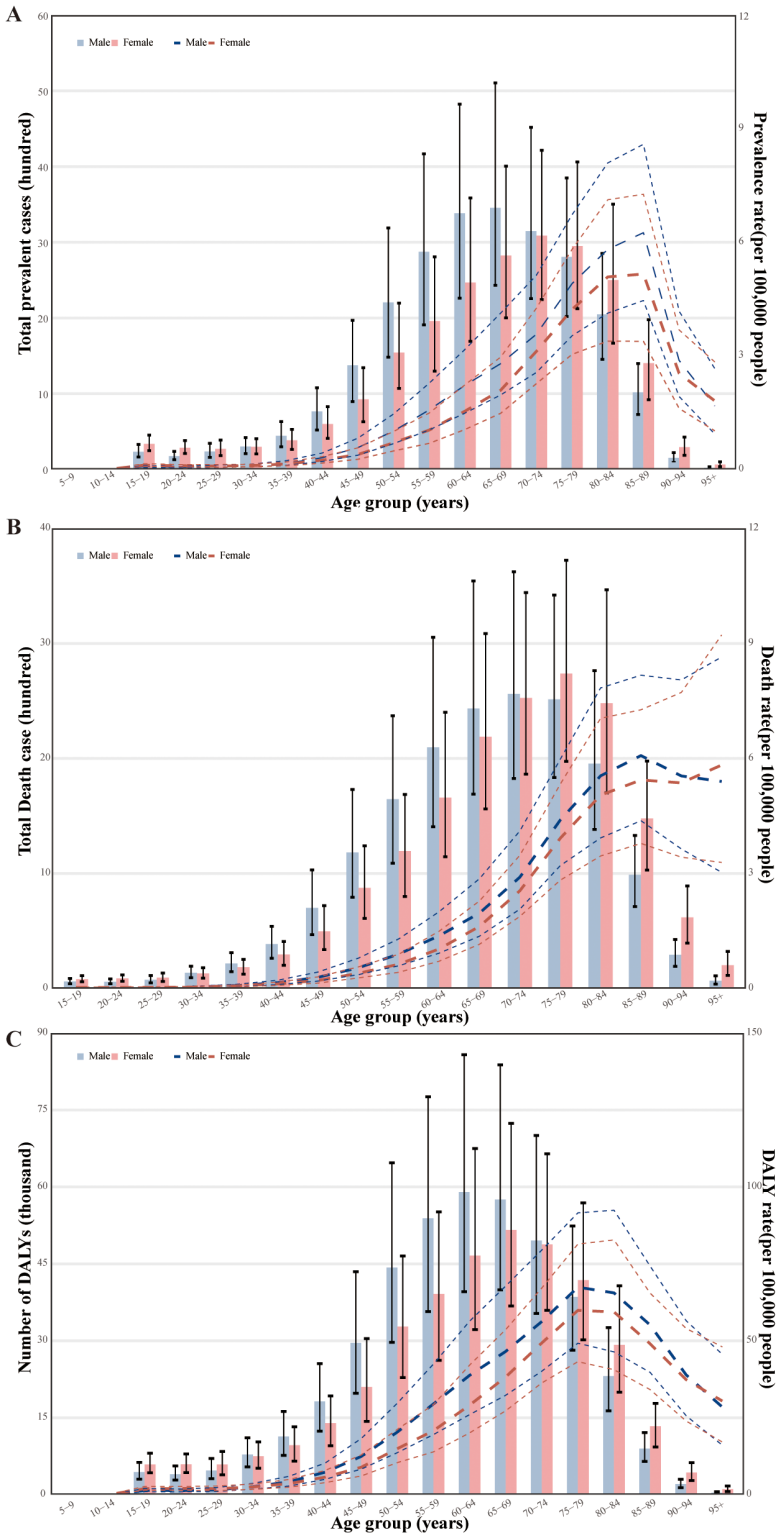
Age-specific numbers and rates of prevalence, deaths, and DALYs attributable to MASH by sex, in 2019. **(A)** Prevalence. **(B)** Deaths. **(C)** DALYs. DALY, disability-adjusted life year.

In 2019, the global point prevalence of MASH-related HCC started to increase in the 30–34 age group and peaked in the 85–89 age group. Similarly, the number of prevalent cases was highest in the 65–69 age group, but then decreased with increasing age. The number of prevalent cases of MASH-related HCC was higher in men up to the 70–74 age group, but MASH-related HCC was more common in women older than 75 years (**Figure 3A**). In 2019, the global MASH-related HCC death rate reached its highest level in the 85–89 age group. In addition, the rate was higher in men across the 15–89 age groups, while they intersected in the 90–94 age groups, and women were higher than men in the highest age group (≥95 years). The number of deaths was highest in the 75–79 age group, for both sexes, after which the numbers decreased with increasing age. The number of deaths caused by MASH-related HCC was higher in men aged up to 70–74 years, while it was higher in women in the 75–79 age group (**Figure 3B**). In both sexes, the global DALY rate of MASH-related HCC increased up to the age of 75–79 years and then decreased with advancing age. This rate was higher in men across all age groups except for the 90+ age group. In addition, the number of DALYs peaked in the 60–64 age group (**Figure 3C**). The number of DALYs due to MASH-related HCC was higher in men up to age 70–74 years, but MASH-related HCC was more common in women older than 75 years.

### 3.3 Association with the sociodemographic index

**Figure 4:**
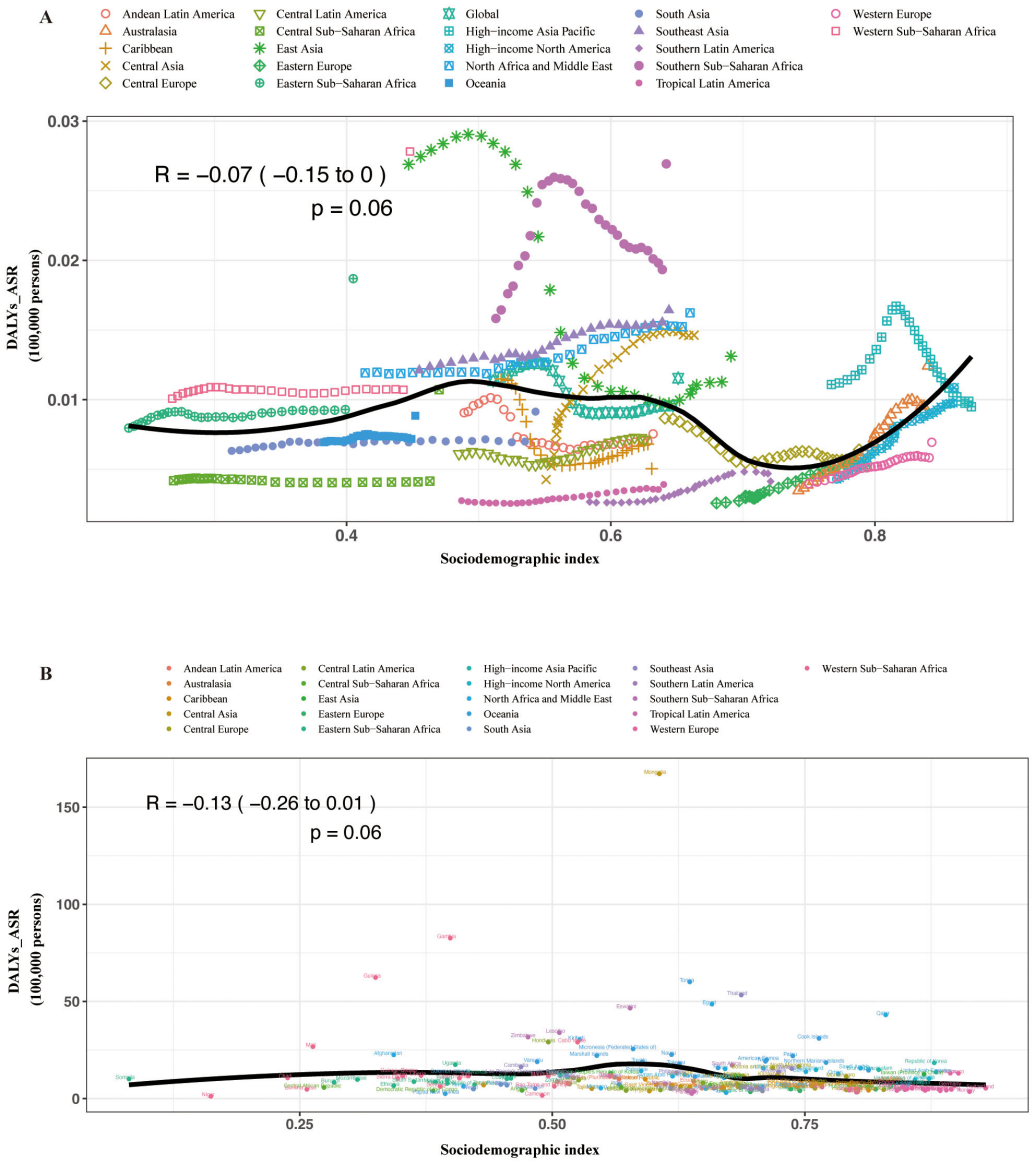
Age-standardized DALY rates attributable to MASH across 21 GBD regions by sociodemographic index for both sexes combined, 1990–2019. **(A)** For each region, points from left to right depict estimates from each year from 1990 to 2019. **(B)** Age-standardized DALY rates attributable to MASH across 195 countries and territories by sociodemographic index for both sexes combined in 2019. DALY, disability-adjusted life year; GBD, Global Burden of Disease Study.

In the regional and national level, we found a reversed V-shaped association between the SDI and the age-standardized DALY rate of MAFLD from 1990 to 2019 (**Supplementary Figures S1A, B**). However, the age-standardized DALY rate of liver cancer due to MASH was not associated with SDI (**Figure 4**).

### 3.4 Risk factors

**Figure 5:**
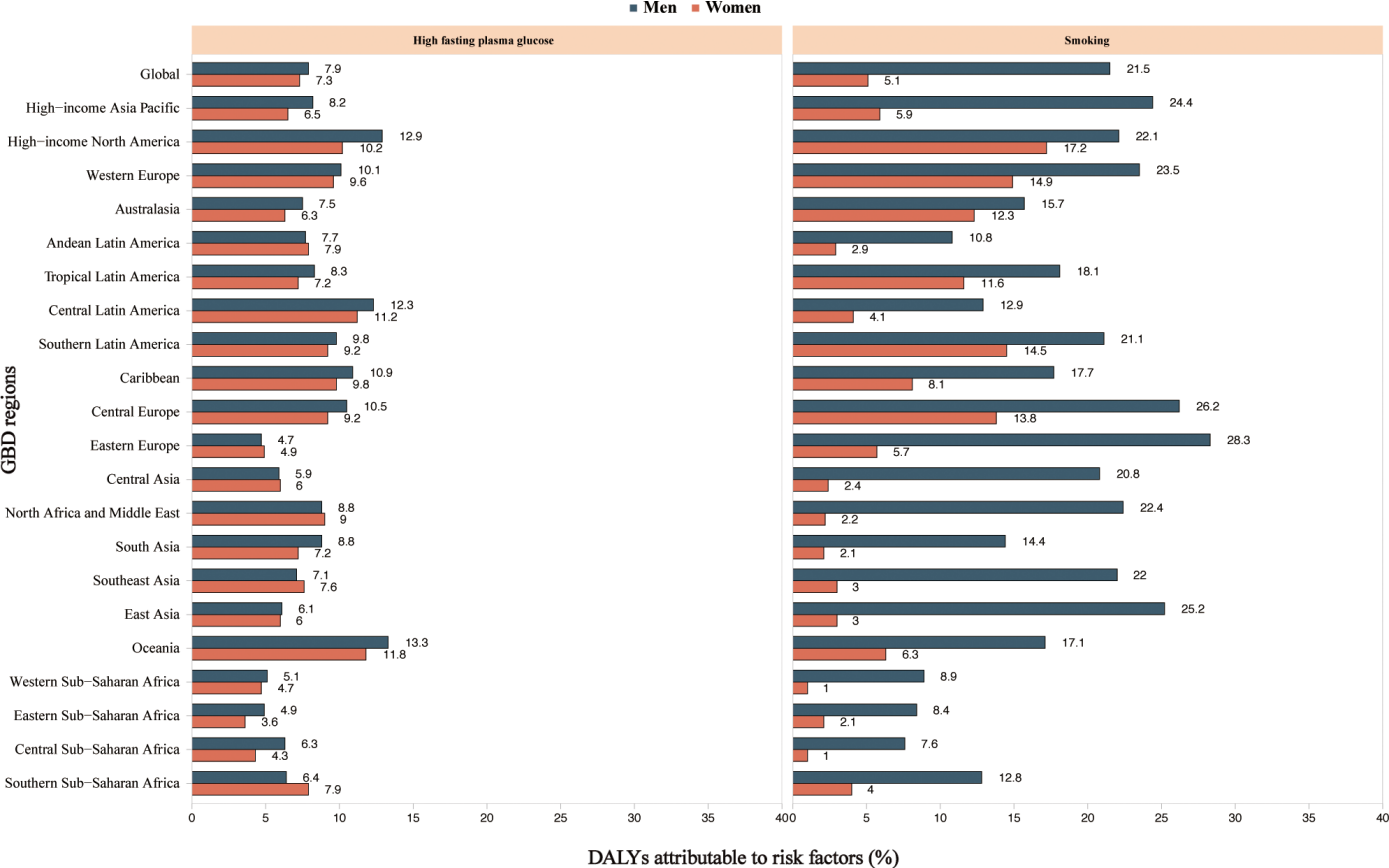
High fasting plasma glucose and smoking age-standardized DALYs attributable to MASH and cancer by region for women and men in 2019. MASH-related HCC. DALY, disability-adjusted life year; GBD, Global Burden of Disease Study.

The proportion of DALYs due to MASH-related HCC attributable to individual risk factors differed across the GBD regions. Globally, high fasting plasma glucose and smoking contributed to DALYs due to MASH-related HCC (**Figure 5**). The proportion of DALYs due to MASH-related HCC attributable to these two risk factors was higher in men. The proportion of smoking as a risk factor was generally higher than that of high fasting plasma glucose.

### 3.5 Bayesian age–period–cohort analysis prediction model

**Figure 6:**
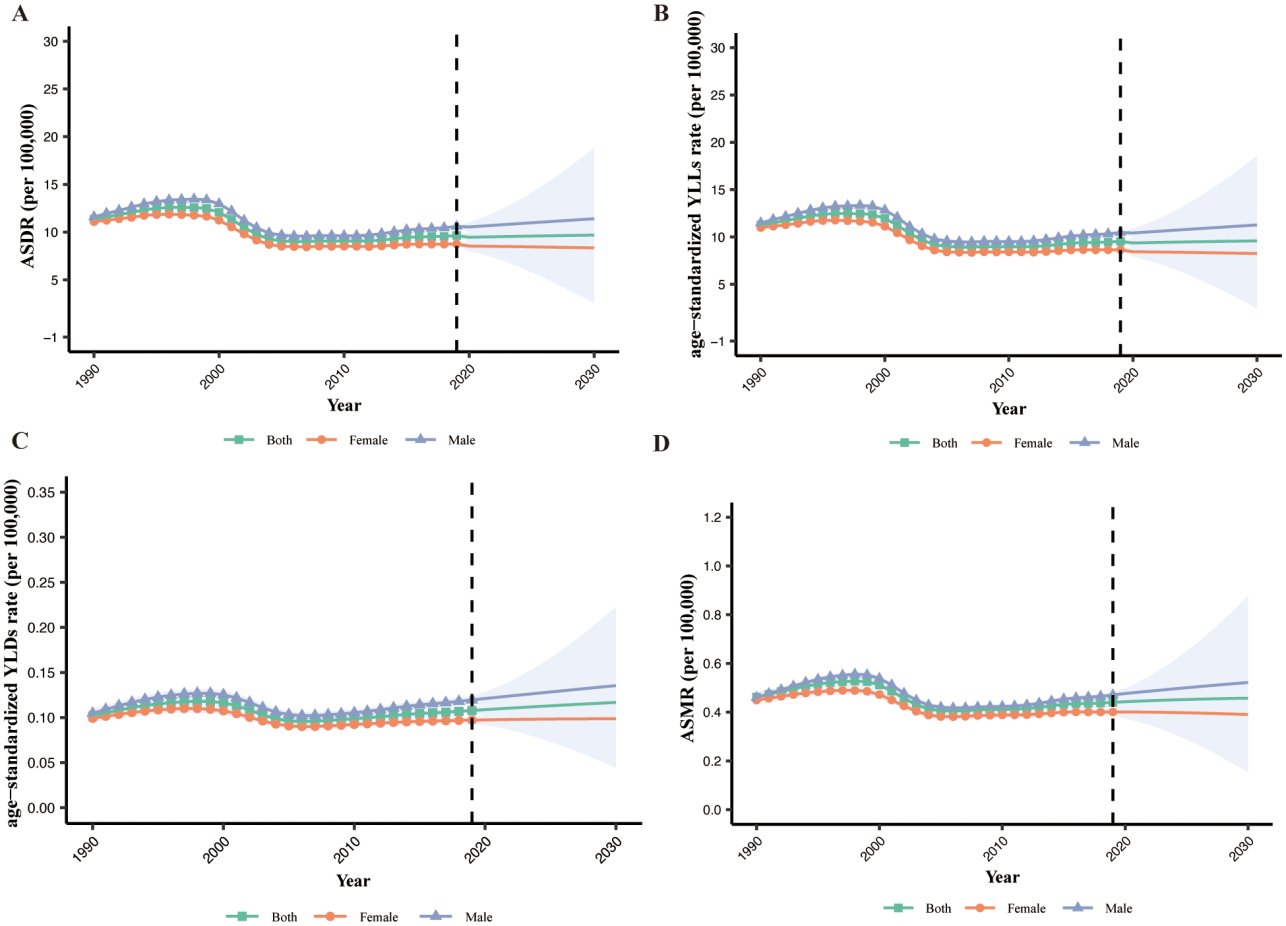
The global burden of MASH-related HCC. **(A)** ASDR by sex from 1990 to 2030. **(B)** YLLs by sex from 1990 to 2030. **(C)** YLDs by sex from 1990 to 2030. **(D)** ASMR by sex from 1990 to 2030. ASDR, age-standardized disability-adjusted life years rate; YLLs, years of life lost; YLDs, years lived with disability; ASMR, age-standardized mortality rate.

Globally, the MASH-related HCC age-standardized disability-adjusted life years rate (ASDR) showed a downward trend from 1990 [11.34 patients per 100,000 (95% CI: 11.32 to 11.36)] to 2019 [9.63 patients per 100,000 (95% CI: 9.62 to 9.64)] (**Figure 6A**). When analyzing the age-standardized years lived with disability (YLDs) rate and age-standardized years of life lost (YLLs) rate separately, we observed a decrease in YLLs rate (−15.3%) but a slight increase in YLDs rate (6.0%) (**Figures 6B, C**). ASDR, age-standardized YLDs rate, and age-standardized YLLs rate will continue to rise slowly over the next decade. Additionally, the MASH-related HCC age-standardized mortality rate (ASMR) (−4.7%) showed a downward trend that is not significant. Based on the projection, the ASDR and ASMR are expected to continue to slowly increase over the next 10 years (**Figure 6D**). Men had higher ASDR and ASMR, with a stable trend, than women. The only modest downward trend observed in ASMR is likely to increase over the next decade. This result suggests that MASH-related HCC burden has been and will be greater among men than among women.

## 4 Discussion

The prevalence of MASH-related HCC is on the rise; however, there has been a concurrent decline in both the mortality rate and the disability-adjusted life years (DALY) rate associated with the condition. Despite this, the total number of cases and absolute mortality figures have increased. This trend suggests several contributing factors, including population growth, demographic aging, enhanced diagnostic capabilities leading to improved early detection, and advancements in palliative care and survivorship support. Advances in diagnostics and widespread screening could lead to earlier identification of cancer cases, contributing to an increased prevalence rate. Early detection often results in a better prognosis and potentially lower mortality rates. Improvements in palliative care and support for survivors can lead to a reduction in DALY rates by alleviating symptoms and improving the quality of life, even for terminal patients. However, the predicted increase in disease burden in the next 10 years may be related to the increasing prevalence of MAFLD and MASH-related HCC. It should be noted that the GBD project uses an estimation or modeling process and does not report real data on the MASH-related HCC burden. This process may underestimate the actual burden of MASH-related HCC and thus may lead to wrong policy decisions.

The increased burden of disease may have several implications for the future: Increased healthcare demand: An increasing number of cases will likely place greater demand on healthcare systems for cancer screening, treatment, and ongoing care. Focus on survivorship: There will be greater emphasis on the quality of life for cancer survivors, including managing long-term effects of cancer and its treatment. Economic impact: The increasing incidence might lead to higher healthcare costs, impacting both individuals and healthcare systems economically. In our study, the total burden of deaths associated with MASH-related HCC was observed to increase with age before the age of 85–89 years. Southern Sub-Saharan Africa, Southeast Asia, North Africa and the Middle East, and Central Asia have the highest age-standardized death rates. This is particularly true in tropical regions of Southern Sub-Saharan Africa and Southeast Asia, where there is frequent exposure to aflatoxin B1, a mycotoxin produced by the *Aspergillus* fungus. These factors could be contributing to the higher mortality rates associated with MASH-related HCC (16). Other possible reasons may include the rising prevalence of obesity and type 2 diabetes due to changes in diet and lifestyle, and of MAFLD (17, 18).

We observed a lack of concordance between the prevalence and mortality rates in MASH-related HCC when ranking regions. For instance, despite the High-income Asia Pacific exhibiting the highest prevalence of MASH-related HCC, it is ranked fifth in terms of mortality. This difference may be due to better management and treatment of MASH-related HCC in these regions. In addition, the onset of MASH-related HCC is usually insidious, and it is often closely related to chronic liver disease. In resource-limited areas, there is a need to ensure that primary healthcare facilities have an adequate pool of knowledge and diagnostic equipment to improve early detection and increase the chances of successful treatment.

The sudden decrease in MASH-related HCC prevalence and death rates in the 85–89 age group could be attributed to survivorship bias, where individuals who might suffer from more severe MASH-related HCC may die earlier, causing a bias in the observed results (19). Additionally, the demographic shift towards a higher proportion of female patients in the MASH-related HCC population over 75 years supports this observation, aligning with the viewpoints presented by Golabi and colleagues (20).

In our study, the lack of a clear link between the MASH-related HCC burden and societal development could indeed relate to universal genetic predispositions, common lifestyle choices affecting liver health, and global environmental factors.

Globally, high fasting plasma glucose and smoking contributed to DALYs due to MASH-related HCC. The contributions underscore the complex interplay between metabolic disorders and lifestyle choices on public health outcomes. The pathophysiological link between high fasting blood glucose and liver fat accumulation, inflammation, and fibrosis may be driven by insulin resistance, a hallmark of type 2 diabetes and an established risk factor for MASH progression (21). Strategies to mitigate this risk involve both primary and secondary prevention efforts, including promoting healthy dietary habits, regular physical activity, and early detection and management of prediabetic states.

Smoking, on the other hand, has a well-documented carcinogenic effect that could directly and indirectly contribute to the pathogenesis of liver cancer (22). The mechanism might involve oxidative stress, induction of DNA mutations, and promotion of a pro-inflammatory state that exacerbates liver injury and fibrosis (23, 24). Smoking cessation programs are thus an essential component of any public health initiative aimed at reducing the incidence of MASH-related liver cancer (25). The observed regional differences in the proportionate contribution of these risk factors to disease burden suggest that public health strategies need to be tailored to the prevailing socioeconomic, cultural, and environmental factors influencing behavior and health outcomes in different populations. In areas where smoking prevalence is particularly high, tobacco control policies, including taxation, marketing restrictions, and education campaigns, should be prioritized. Furthermore, the greater impact of high fasting blood glucose and smoking on liver cancer DALYs among men highlights a need for sex-specific health interventions and increased awareness of these risk factors in men.

The analysis of the temporal trend in the ASDR for MASH from 1990 to 2019 indicates a decrease in the overall disease burden. This decline, from 11.34 to 9.63 cases per 100,000 population, suggests potential improvements in public health interventions and healthcare accessibility. When disaggregating the ASDR into age-standardized YLDs and YLLs, we observe decoupled trends. The decrease in YLLs rates by 15.3% could be attributed to improvements in early detection, medical treatment, and possibly decreased mortality related to MASH-related HCC. However, the slight increase in YLDs rates by 6.0% merits attention as it indicates a worsening in quality of life or an increase in the prevalence of living with disability due to MASH-related HCC. In our study, the ASDR, age-standardized YLDs rate, and age-standardized YLLs rate will continue to rise slowly over the next decade. This is consistent with other studies (6).This anticipated rise may be linked to aging populations, lifestyle factors, or heightened disease awareness, leading to increased reporting. The consistent higher rates in men versus women require targeted interventions.

## 5 Strengths and limitations of this study

A strength of the study is that we have provided comprehensive estimates of the levels and trends associated with MASH-related HCC and its risk factors at the global, regional, and national levels between 1990 and 2019. Moreover, the BAPC model was used to predict the trend of ASDR and ASMR in the next 10 years. This study has several limitations: (1) The quality of the data is dependent on the original data collection process. Under-registration and underreporting of cancer cases, especially in regions with limited resources, can lead to misdiagnosis or underdiagnosis, impacting the accuracy of our estimates. To address this, we can improve cancer registration systems and use statistical models to adjust for unreported cases. (2) Some risk factors, such as genetic predisposition, although rare, could not be taken into account in our estimations. (3) Many countries lack efficient systems for registering deaths, relying instead on verbal autopsies, which may not be as accurate as medical autopsies. Recall bias and variability in implementation can affect data accuracy. Combining hospital records with health survey data and providing systematic training for verbal autopsy implementers can help mitigate these biases. (4) In our study, we only analyzed the correlation between the burden of MASH-related HCC and social development, lacking the correlation of the disease with prevalent genetic predisposition, common lifestyle choices affecting liver health, and global environmental factors. (5) Moreover, compared to NAFLD, MAFLD is considered a more suitable term at a population level, and using the MAFLD criteria allows for the identification of a greater number of individuals with liver damage. Unfortunately, MASH outcomes are not yet included in the GBD data. Therefore, we used the NASH data from the 2019 GBD database.

## 6 Conclusion

Liver cancer is a major public health problem, and MASH-related HCC similarly requires substantial healthcare and economic costs. Although the death and DALY rates declined during the study period, the corresponding counts are increasing. With prevalence increasing from 1990 to 2019, MASH-related HCC will likely become an even greater problem in the future. The reported global, regional, and national burden of MASH-related HCC and its risk factors could help provide a more accurate projection of the future disease burden.

## Data Availability

The original contributions presented in the study are included in the article/**Supplementary Material**. Further inquiries can be directed to the corresponding author.
