# Supplementary material for: Worldwide burden of liver cancer due to metabolic dysfunction-associated steatohepatitis from 1990 to 2019: insights from the Global Burden of Disease study
Source: Front Oncol. 2024 Aug 29;14:1424155. doi: 10.3389/fonc.2024.1424155 (PMC11390418; doi:10.3389/fonc.2024.1424155)
Supplement: Supplementary file 1 [file DataSheet1.docx]

Supplementary Material

# Supplementary Figures


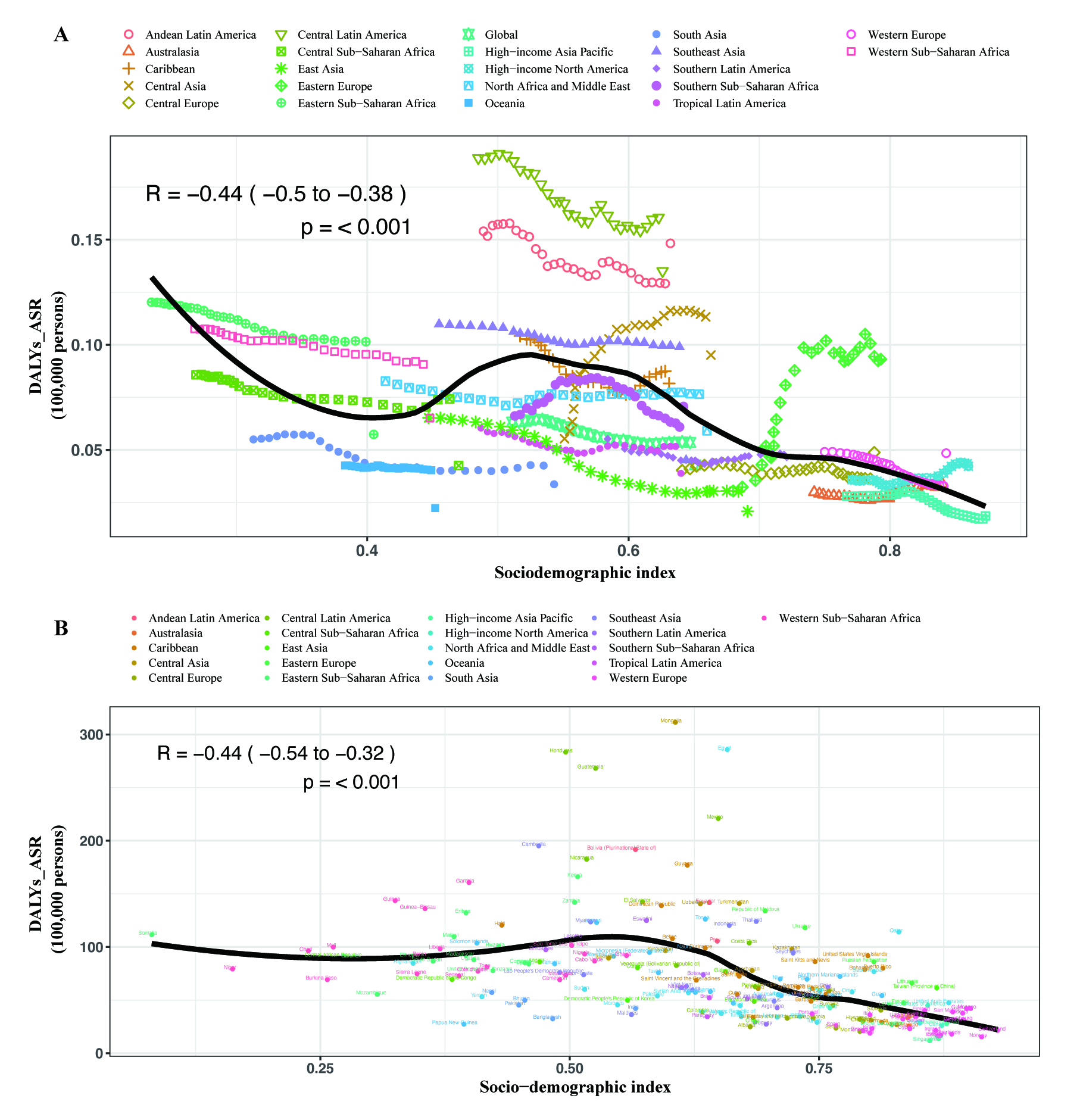


**Supplementary Fig S1.** Age-standardized DALY rates attributable to NAFLD across 21 GBD regions by Socio-demographic Index for both sexes combined, 1990–2019. (A)For each region, points from left to right depict estimates from each year from 1990 to 2019. (B)Age-standardized DALY rates attributable to NAFLD across 195 countries and territories by Socio-demographic Index for both sexes combined in 2019. DALY, disability-adjusted life year; GBD, Global Burden of Disease Study.
